# Development of a deep learning model that predicts critical events of pediatric patients admitted to general wards

**DOI:** 10.1038/s41598-024-55528-1

**Published:** 2024-02-27

**Authors:** Yonghyuk Jeon, You Sun Kim, Wonjin Jang, June Dong Park, Bongjin Lee

**Affiliations:** 1 Department of Pediatrics, Seoul National University College of Medicine, Seoul National University Hospital, 101, Daehak-ro, Jongno-gu, Seoul, 03080 Korea; 2 Department of Pediatrics, National Medical Center, Seoul, Republic of Korea; 3 Innovative Medical Technology Research Institute, Seoul National University Hospital, Seoul, Republic of Korea

**Keywords:** Medical research, Risk factors

## Abstract

Early detection of deteriorating patients is important to prevent life-threatening events and improve clinical outcomes. Efforts have been made to detect or prevent major events such as cardiopulmonary resuscitation, but previously developed tools are often complicated and time-consuming, rendering them impractical. To overcome this problem, we designed this study to create a deep learning prediction model that predicts critical events with simplified variables. This retrospective observational study included patients under the age of 18 who were admitted to the general ward of a tertiary children’s hospital between 2020 and 2022. A critical event was defined as cardiopulmonary resuscitation, unplanned transfer to the intensive care unit, or mortality. The vital signs measured during hospitalization, their measurement intervals, sex, and age were used to train a critical event prediction model. Age-specific z-scores were used to normalize the variability of the normal range by age. The entire dataset was classified into a training dataset and a test dataset at an 8:2 ratio, and model learning and testing were performed on each dataset. The predictive performance of the developed model showed excellent results, with an area under the receiver operating characteristics curve of 0.986 and an area under the precision-recall curve of 0.896. We developed a deep learning model with outstanding predictive power using simplified variables to effectively predict critical events while reducing the workload of medical staff. Nevertheless, because this was a single-center trial, no external validation was carried out, prompting further investigation.

## Introduction

Early detection of deteriorating patients is crucial in order to provide timely intervention before critical events, such as cardiopulmonary resuscitation (CPR), take place. Cardiac arrest due to respiratory failure is known to be more common in children compared to adults, whereas cardiac arrest of cardiac origin is relatively rare in children^1–3^. For this reason, pediatric patients may have a higher chance of receiving intervention before cardiac arrest occurs. The pediatric early warning score (PEWS) is one of the means that has been developed in an effort to recognize deteriorating patients as early as possible^4–6^.

PEWS determines a patient’s risk level by measuring and scoring several vital sign values, such as blood pressure (BP) and heart rate (HR) by age. A few examples of PEWS include the Bedside PEWS, the Brighton PEWS, the Melbourne Activation Criteria, and the Bristol PEWS^5–11^. In initial studies, these methods demonstrated very high predictive performance, with an area under the receiver operating characteristic curve (AUROC) of around 0.9^4,6^. However, numerous validation studies on different types of PEWS carried out by multiple institutions were unable to replicate the same outcomes and showed relatively low performance (AUROC 0.62–0.86)^9,10,12–14^. In addition, the process of obtaining necessary parameters and calculating these scores demands considerable time and effort from medical staff, and the qualitative data required in scoring PEWS, such as capillary refill time and respiratory effort, are often not readily available in contrast to easily obtainable values such as BP, HR, and respiratory rate (RR)^4,6^.

As a way to compensate for these shortcomings, the application of machine learning, particularly deep learning, in constructing predictive models has been drawing attention in the research field. Most studies were conducted on adults, and only a few examined pediatric models. One retrospective study assessed a model that used 29 variables to predict the likelihood of transmission to the intensive care units (ICU) within 24 h, and the AUROC was 0.912 (95% confidence interval [CI] 0.905–0.919). Although the accuracy of the predictions was excellent, it might be impractical to collect and analyze 29 variables^15^. In another retrospective study of pediatric subjects, a long short-term memory (LSTM) model with a promising AUROC of 0.923 was developed using fewer parameters. The LSTM model, however, could only be used when there are more than 20 consecutive time-stamped vital sign data points. Consequently, initial prediction in general wards can be challenging because vital signs are not typically recorded frequently, unlike in ICU^16^.

Therefore, the authors designed this study to create a deep learning model that can anticipate crucial events utilizing simplified variables without long-term continuous measurement values.

## Methods

### Study setting and data source

This retrospective cross-sectional observational study was conducted at a tertiary children’s hospital with about 350 beds. The subjects were patients under the age of 18 who had been admitted to the general ward of the children’s hospital between January 2020 and December 2022. The pseudonymized data used for analysis were collected from the clinical data warehouse of the hospital information system. The measurements of Systolic BP (SBP), diastolic BP (DBP), HR, RR, body temperature (BT), and the oxygen saturation measured with pulse oximetry (SpO_2_) were recorded in the general ward. Measurements from the emergency department or ICU were excluded. Recorded time, sex, age (in months), admission date, discharge date, and pseudonymized study-specific identification code were collected.

This study was approved for exemption from review by the Institutional Review Board of Seoul National University Hospital because it used only pseudonymized information and did not collect personally identifiable information (H-2209-001-1032). Since only information that could not identify the research subjects was used, the above committee confirmed that it was impossible to obtain consent from specific subjects. Moreover, the study was conducted in accordance with the principles of the Declaration of Helsinki.

### Data preprocessing

The pseudonymized identification code and hospitalization date were combined to create a unique classification code according to each individual hospitalization date, which was defined as the individual hospitalization identification code (IHID). The collected data were classified according to IHID, sorted in ascending order of vital sign measurement time, and missing values among SBP, DBP, HR, RR, BT, and SpO_2_ were replaced with the immediately preceding values. In addition, the interval of vital sign measurement time was calculated within the same IHID (each vital sign measurement time—previous measurement time, in minutes), and this was defined as the measurement interval. Since the normal ranges of BP, HR, and RR in children differ according to age, z-scores for each age were calculated and used for analysis. Centile charts of vital signs for each age developed in a previous study were used for z-score conversion^17^.

Critical events were defined as cases where CPR occurred in the general ward, unexpected transfers to the ICU, and cases of mortality (results of CPR or discontinuation of life-sustaining treatment)^18,19^. Critical records were defined as the data measured from 6 h before the occurrence of the critical event to the time of occurrence in the case of unexpected ICU transfer or mortality, and in the case of CPR, it was defined as the data measured from 6 h before the occurrence to 30 min after the occurrence (from 6 h before CPR until death in the case of mortality after CPR). In order to perform deep learning on critical records, the total records were divided into two groups: critical group and non-critical group. Since the records of individuals who experienced a critical event will have a mixture of critical records and non-critical records, IHID’s non-critical records with critical events were excluded from the non-critical group. In addition, since it is expected to be an imbalanced dataset in which the size of the non-critical group is substantially larger than the sample size of the critical group, only the last records for each IHID among the non-critical groups were used for deep learning. In general, it is common sense that vital sign records measured during hospitalization for each IHID are not limited to just one occurrence but rather numerous. Therefore, we anticipated that retaining only the last record per IHID among the vital sign records in the non-critical group, and utilizing all records in the critical group, would relatively alleviate the imbalance between the two groups. R version 4.3.1 (R Foundation for statistical computing, Vienna, Austria; https://www.r-project.org) was used for data preprocessing, and open packages such as the generalized additive models for location scale and shape and sitar were used in this process^20–22^.

### Deep learning and data analysis

The preprocessed dataset was divided into a training set and a test set at a ratio of 8:2, and each was used for model training and testing. A simple artificial neural network (ANN) algorithm based on the multilayer perceptron was used for deep learning. Nine parameters used for learning were age, sex, z-score of SBP, z-score of DBP, z-score of HR, z-score of RR, BT, SpO_2_, and the measurement interval. The above features were normalized to a value between 0 and 1. The ANN model was composed of 3 hidden layers (each with node counts of 128, 128, and 64, respectively), and a 30% dropout was applied after each layer. The Adam optimizer and rectified linear unit activator were used in the process^23^. It was trained for 10,000 epochs with a learning rate of 0.0001 using Python version 3.8.10 (Python Software Foundation, Beaverton, OR, USA; https://www.python.org). Scikit-learn library was used for normalization^24^, PyTorch was used for model training and test^25^, and matplotlib and Shapley additive explanation (SHAP) library were used for visualization^26^. Since the measurement interval value of the first record for each IHID cannot be calculated (missing value), the average value of all measurement intervals was imputed. Continuous variables were described as median (interquartile range) and categorical variables as number (%).

### Outcomes

The primary outcome of this study was the overall predictive performance of the developed model. Accuracy, AUROC, and area under the precision-recall curve (AUPRC) were used to evaluate the predictive performance of the model. The secondary outcomes included subdividing critical events into CPR occurrence, unexpected ICU transfer, and mortality, respectively, and evaluating the performance of the developed model for each. Additionally, based on the time elapsed before a critical incident occurred, measurements were divided into six subgroups: 0–1 h, 1–2 h, 2–3 h, 3–4 h, 4–5 h, and 5–6 h. For each subgroup, the predictive performance of the model was included. It also included an assessment of the importance of the prediction process for each feature used in learning and the correlation between features.

## Results

### Baseline characteristics

During the study period, 13,787 patients were hospitalized a total of 22,184 times, and 1,039,070 vital sign records were analyzed. When analyzed by IHID, the age at admission was 69.0 (23.0–135.0) months, and 9,485 (42.8%) were girls. The duration of hospitalization was 3.0 (2.0–7.0) days.

Of the total records of vital signs, 632 (0.1%) cases were critical records, and the median measurement interval was 161.0 min. Detailed descriptions of SBP, DBP, HR, RR, BT, and SpO_2_ are summarized in Table 1. There were 14,227 records remaining after data preprocessing; the age was 74.0 (22.0–139.0) months, and 6,041 (42.5%) were girls. The critical group included 632 (4.4%) of the patients, and among the critical records, 261 instances involved CPR, 238 cases involved unplanned ICU transfers, and 141 cases involved fatalities. There were 8 records of patients who died as a result of CPR. Additional information is described in greater depth in Table 2. The calculated mean value for imputing missing data in the first measurement interval for each IHID was 276.17.

**Table 1:** Baseline characteristics of all vital sign records.

Variables	Vital sign records (n = 1,039,070)
Critical records	632 (0.1)
Measurement intervals (minutes)	161.0 (43.0–245.0)
Systolic blood pressure	
Measured value (mmHg)	104.0 (95.0–115.0)
Z-score by age*	− 0.3 (− 0.7 to 0.1)
Diastolic blood pressure	
Measured value (mmHg)	64.0 (55.0–73.0)
Z-score by age*	0.1 (− 0.3 to 0.6)
Heart rate	
Measured value (beats/minute)	108.0 (91.0–126.0)
Z-score by age*	− 0.9 (− 1.4 to − 0.3)
Respiratory rate	
Measured value (breaths/minute)	24.0 (20.0–30.0)
Z-score by age*	− 0.4 (− 0.6 to − 0.1)
Body temperature (℃)	36.8 (36.5–37.2)
Oxygen saturation (%)	99.0 (97.0–100.0)

Values are presented as median (interquartile range) or number (%).

*The centile chart developed in the previous paper was used to calculate the z-score by age.

**Table 2:** Characteristics of datasets used to develop deep learning models.

Variables	Total (n = 14,227)	Non-critical group (n = 13,595)	Critical group (n = 632)
Age (months)	74.0 (22.0–139.0)	75.0 (23.0–140.0)	64.0 (10.5–124.5)
Female	6041 (42.5)	5766 (42.4)	275 (43.5)
VS measurement intervals (minutes)	240.0 (162.0–480.0)	240.0 (177.0–480.0)	7.0 (2.0–45.0)
Systolic blood pressure			
Measured value (mmHg)	104.0 (95.0–113.0)	104.0 (96.0–113.0)	101.0 (89.0–116.5)
Z-score by age*	− 0.4 (− 0.7 to 0.0)	− 0.4 (− 0.7 to 0.0)	− 0.4 (− 0.9 to 0.2)
Diastolic blood pressure (mmHg)			
Measured value (mmHg)	63.0 (55.0–71.0)	63.0 (55.0–70.0)	61.0 (50.0–72.5)
Z-score by age*	0.1 (− 0.3 to 0.5)	0.1 (− 0.3 to 0.5)	0.1 (− 0.5 to 0.7)
Heart rate			
Measured value (beats/minute)	103.0 (88.0–123.0)	103.0 (88.0–121.0)	126.0 (80.0–160.0)
Z-score by age*	− 1.0 (− 1.4 to − 0.5)	− 1.0 (− 1.4 to − 0.5)	− 0.3 (− 1.7 to 0.8)
Respiratory rate			
Measured value (breaths/minute)	24.0 (20.0–28.0)	24.0 (20.0–28.0)	33.0 (21.0–44.0)
Z-score by age*	− 0.5 (− 0.6 to − 0.3)	− 0.5 (− 0.6 to − 0.3)	0.1 (–0.6 to 0.9)
Body temperature (℃)	36.7 (36.5–37.0)	36.7 (36.5–37.0)	36.9 (36.5–37.6)
Oxygen saturation (%)	99.0 (98.0–100.0)	99.0 (98.0–100.0)	88.0 (61.0–98.0)
Critical event			
CPR	261 (1.8)	0 (0.0)	261 (41.3)
ICU transfer	238 (1.7)	0 (0.0)	238 (37.7)
Mortality	141 (1.0)	0 (0.0)	141 (22.3)

Values are presented as median (interquartile range) or number (%).

VS, vital sign; CPR, cardiopulmonary resuscitation; ICU, intensive care unit.

*The centile chart developed in the previous paper was used to calculate the z-score by age.

### Main outcomes

**Figure 1:**
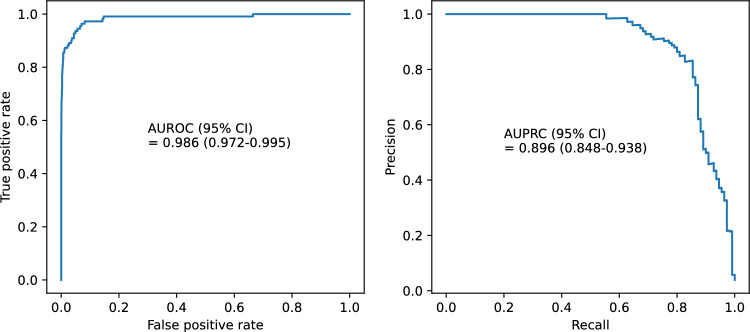
Receiver operating characteristic curve and precision-recall curve. AUROC = area under the receiver operating characteristic curve, AUPRC = area under the precision-recall curve.

**Figure 2:**
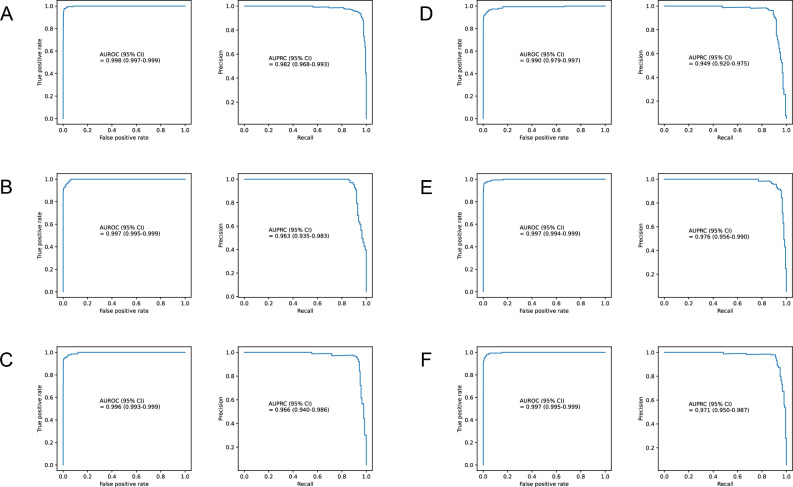
Performance of the developed model according to the time interval before a critical event occurs. AUROC, AUPRC (**A**) 0–1 h, (**B**) 1–2 h, (**C**) 2–3 h, (**D**) 3–4 h, (**E**) 4–5 h, and (**F**) 5–6 h before a critical event occurs. AUROC = area under the receiver operating characteristic curve, AUPRC = area under the precision-recall curve.

The accuracy of the developed model was 0.988, AUROC (95% CI) was 0.986 (0.972–0.995), and AUPRC (95% CI) was 0.896 (0.848–0.938) (Fig. 1). In the performance evaluation for each detailed item of the critical events, the detailed item, AUROC (95% CI), AUPRC (95% CI) are respectively as follows: CPR occurrence, 0.967 (0.928–0.988), 0.451 (0.322–0.585) (Supplementary Fig. S1); unexpected ICU transfer, 0.964 (0.951–0.975), 0.203 (0.139–0.276) (Supplementary Fig. S2); and mortality, 0.995 (0.993–0.997), 0.683 (0.551–0.809) (Supplementary Fig. S3). In subgroup evaluation by time interval, the time interval, AUROC, and AUPRC of each time subgroup are as follows: 0–1 h, 0.998, 0.982; 1–2 h, 0.997, 0.963; 2–3 h, 0.996, 0.966; 3–4 h, 0.990, 0.949; 4–5 h, 0.997, 0.976; and 5–6 h, 0.997, 0.971. The respective 95% Cis and graphical illustrations are shown in Fig. 2.

**Figure 3:**
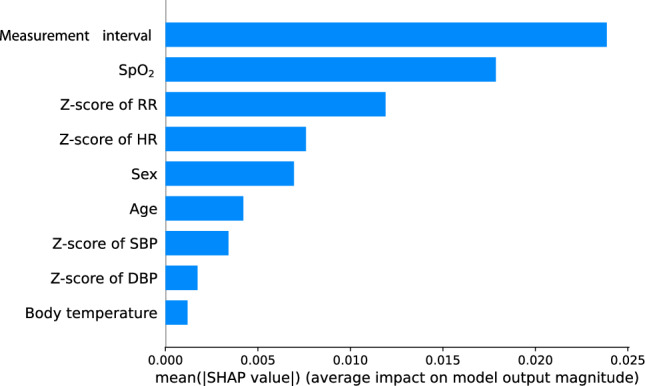
Impact on the output of each variable used in the model. The higher the mean SHAP value (the longer the blue bar to the right), the greater the impact on the predictive model. SpO_2_ = oxygen saturation, RR = respiratory rate, HR = heart rate, SBP = systolic blood pressure, DBP = diastolic blood pressure, SHAP = shapley additive explanations.

**Figure 4:**
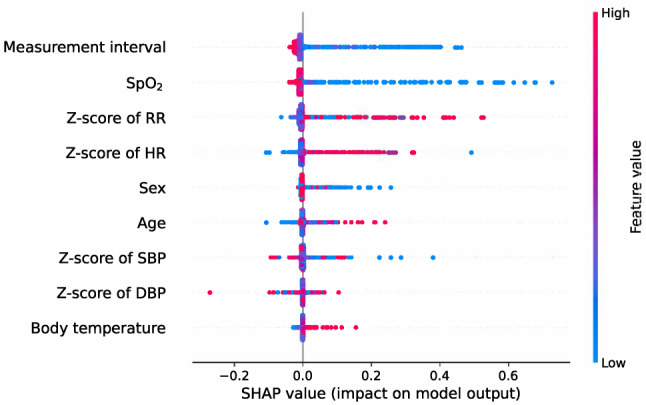
SHAP values for each feature used in the model. Shows the change in the impact value for the model output depending on whether the value of each feature is high (red) or low (blue). For example, when the measurement interval is low (blue), the SHAP value is higher than when the measurement interval is high (red), thus it can be interpreted that a short measurement interval is important in predicting the patient’s deterioration. SpO_2_ = oxygen saturation, RR = respiratory rate, HR = heart rate, SBP = systolic blood pressure, DBP = diastolic blood pressure, SHAP = shapley additive explanations.

Among the features used to predict the outcomes, measurement interval had the highest impact, followed by SpO_2_ and a z-score of RR (Fig. 3). How the model prediction impact changes according to the high and low values of each feature is shown in Fig. 4. The lower the measurement interval (blue), the higher the impact on the model output, and the higher the measurement interval (red), the lower the impact. SpO_2_ also showed the same pattern as the measurement interval. On the other hand, greater z-scores for RR and HR had a greater impact on outcomes, while lower z-scores for RR and HR had a lesser effect on outcomes (Fig. 4).

The correlation between the features was studied to further characterize the model. The SHAP value (the impact of the model output) increased with a smaller measurement interval, as in the prior results, but this time around, the z-score of HR had no discernible impact on the value (Supplementary Fig. S4). Regardless of whether the measurement interval was high or low, SpO_2_ and SHAP values consistently had an inverse correlation, and this tendency was more pronounced when the measurement interval was smaller (Supplementary Fig. S5). The supplementary figures provide a summary of the inter-feature influence of parameters that are not mentioned above (z-score of RR, Supplementary Fig. S6; z-score of HR, Supplementary Fig. S7; sex, Supplementary Fig. S8; age, Supplementary Fig. S9; z-score of SBP, Supplementary Fig. S10; z-score of DBP, Supplementary Fig. S11; and body temperature, Supplementary Fig. S12).

## Discussion

Through this study, we created a deep learning model that uses simplified variables, including vital signs, age, sex, and measurement interval, to predict the need for intervention in pediatric patients who are deteriorating. Our approach, in contrast to earlier studies, predicts the probability of transfer to the ICU using only a handful of variables without the need for accumulated measurements. Furthermore, the model demonstrated an AUROC of 0.986 and an AUPRC of 0.896, which were significantly better than those of earlier studies^15,16^.

Numerous studies on previously developed PEWS have reported outstanding AUROC values of around 0.9, but the process of collecting and calculating the parameters for the scoring system is complex and time-consuming, which can significantly increase the workload of the medical staff. Even when the efficacy of the prediction model is high, its impracticality can become an obstacle in clinical settings. It is important to evaluate the workload of medical staff, especially in an environment with limited medical resources^27–29^. The prediction model created in this study can decrease such workload for the medical staff because it utilizes vital signs, sex, and age as parameters that are expressed in plain values and are easy to access because they are collected in the hospital electronic medical record system. Moreover, predictions with a deep learning model can be generated automatically without manually entering values into a computer, which can eliminate the workload of the medical staff entirely.

In this investigation, the measurement interval was used as a learning parameter as opposed to the LSTM model study, which needs consecutive measurement results. Vital signs are typically not monitored as regularly in general wards as they are in ICUs, but the frequency increases if a patient’s condition deteriorates. We were able to create a prediction model without the necessity of 20 consecutive observations because our prediction model was built to reflect this idea. As a result, predictions can be made before a collection of subsequent measurements is complete.

In the detailed analysis of critical events, AUROC consistently exceeded 0.96 for all CPR occurrences, ICU transfers, and deaths, mirroring the performance in predicting overall critical events. However, AUPRC exhibited a notable decline, possibly stemming from the model's lack of specialized training for individual events. Subsequent subgroup analysis by time interval yielded unexpected results. Contrary to expectations, proximity to critical events did not necessarily enhance prediction performance. Remarkably, the model demonstrated superior results across all time periods compared to the overall critical events prediction. The black box nature of deep learning made it challenging for the authors to provide a definitive explanation for these results. Yet, upon reflection, it was noted that the model was developed without the intention of making predictions based on a series of continuous measurements; instead, it analyzed only measurements from a single timestamp. Another crucial point to consider was that the parameters used for learning did not incorporate information capable of estimating the time from measurement to event occurrence, which was deemed a significant explanatory factor.

The persisting question surrounded the superior results observed in the time-specific subgroups compared to the overall performance. It was hypothesized that as measurements corresponding to critical events were divided into subgroups, the imbalance between the non-critical group and critical subgroups increased, thus maintaining an excellent AUROC. Additionally, to explain the enhanced AUPRC, the authors considered the homogeneity of the data. The non-critical group in the study comprised the last vital sign measurements taken before discharge from patients without a critical event, making it a relatively stable and homogeneous group. Conversely, the critical group, subject to medical interventions, naturally exhibited diversity in collected measurement values. It was reasoned that the longer the collection time, the greater the diversity, and narrowing the collection time window would decrease this diversity. Therefore, as the time window for measurement value collection narrowed, the homogeneity of the collected measurement values increased. Even if measurements at 5–6 h were relatively stable compared to those at 0–1 h, the existence of characteristics clearly distinguishable from the non-critical group just before discharge could contribute to elevated AUROC and AUPRC levels. Still, it is crucial to acknowledge that this explanation is rooted in assumptions and hypotheses, lacking concrete, objective evidence. Therefore, the interpretation and judgment of these findings are ultimately left to the readers.

This study has several limitations. The first is that no external validation was done, as the study was only conducted at one center. During the early stages of development, the PEWS performed outstandingly, but validation tests conducted in diverse settings had mixed results. Although the AUROC and AUPRC of our predictive model were high, we cannot ensure that the performance can be duplicated in other hospitals or in other target populations, as in the case of PEWS. Although overfitting was minimized by applying a 30% dropout to each layer, the possibility of overfitting the dataset in this study cannot be ruled out. Therefore, it is necessary to conduct follow-up studies for external validation in collaboration with other hospitals. Another limitation is that in the first measurement for each IHID, the measurement interval is inevitably missing, and in this case, the average value of the entire measurement interval was replaced. Considering that the factor with the most influence on our predictive model is the measurement interval (Fig. 3), it may be difficult to guarantee its performance for predictive power with only the first measurement. However, the total measurement interval was 240 (162.0–480.0) minutes (Table 2), and the SHAP value changed rapidly when the measurement interval was low (Fig. 4). Therefore, the possibility of significantly changing the risk can be considered sufficiently low even if the average value of the interval was used for the first measurement taken in the general ward. In addition, an essential aspect to address in this study is that, even though deep learning models exhibit proficiency in predicting critical events, it is imperative to closely monitor a patient's organ function preceding major occurrences such as CPR or mortality. Despite the strong predictive capabilities of these models, the meticulous monitoring of a patient's organ function by medical staff remains indispensable for gaining insights into the patient's dynamic health status, allowing timely interventions and personalized care. We believe that the synergistic use of predictive models and continuous monitoring can ensure a comprehensive and proactive approach to patient care in critical situations.

## Conclusion

Herein, we developed a deep learning model that predicts critical events using simplified variables. The performance of the model was excellent and worked without consequential serial measurements. A well-designed follow-up multicenter study is needed for external validation.

### Supplementary Information

Supplementary Figures.

## Data Availability

The datasets generated during and/or analyzed during the current study are available from the corresponding author on reasonable request.
