# Supplementary material for: Development of a deep learning model that predicts critical events of pediatric patients admitted to general wards
Source: Sci Rep. 2024 Feb 27;14:4707. doi: 10.1038/s41598-024-55528-1 (PMC10897152; doi:10.1038/s41598-024-55528-1)
Supplement: Supplementary file 1 — Supplementary Figures. [file 41598_2024_55528_MOESM1_ESM.docx]

**Supplementary information**

**Figure S1**. Receiver operating characteristic curve and precision-recall curve for CPR occurrence prediction of the developed model.

AUROC = area under the receiver operating characteristic curve, AUPRC = area under the precision-recall curve, CPR = cardiopulmonary resuscitation.

**Figure S2**. Receiver operating characteristic curve and precision-recall curve for unexpected ICU admission prediction of the developed model.

AUROC = area under the receiver operating characteristic curve, AUPRC = area under the precision-recall curve, ICU = intensive care unit.

**Figure S3**. Receiver operating characteristic curve and precision-recall curve for mortality occurrence prediction of the developed model.

AUROC = area under the receiver operating characteristic curve, AUPRC = area under the precision-recall curve.


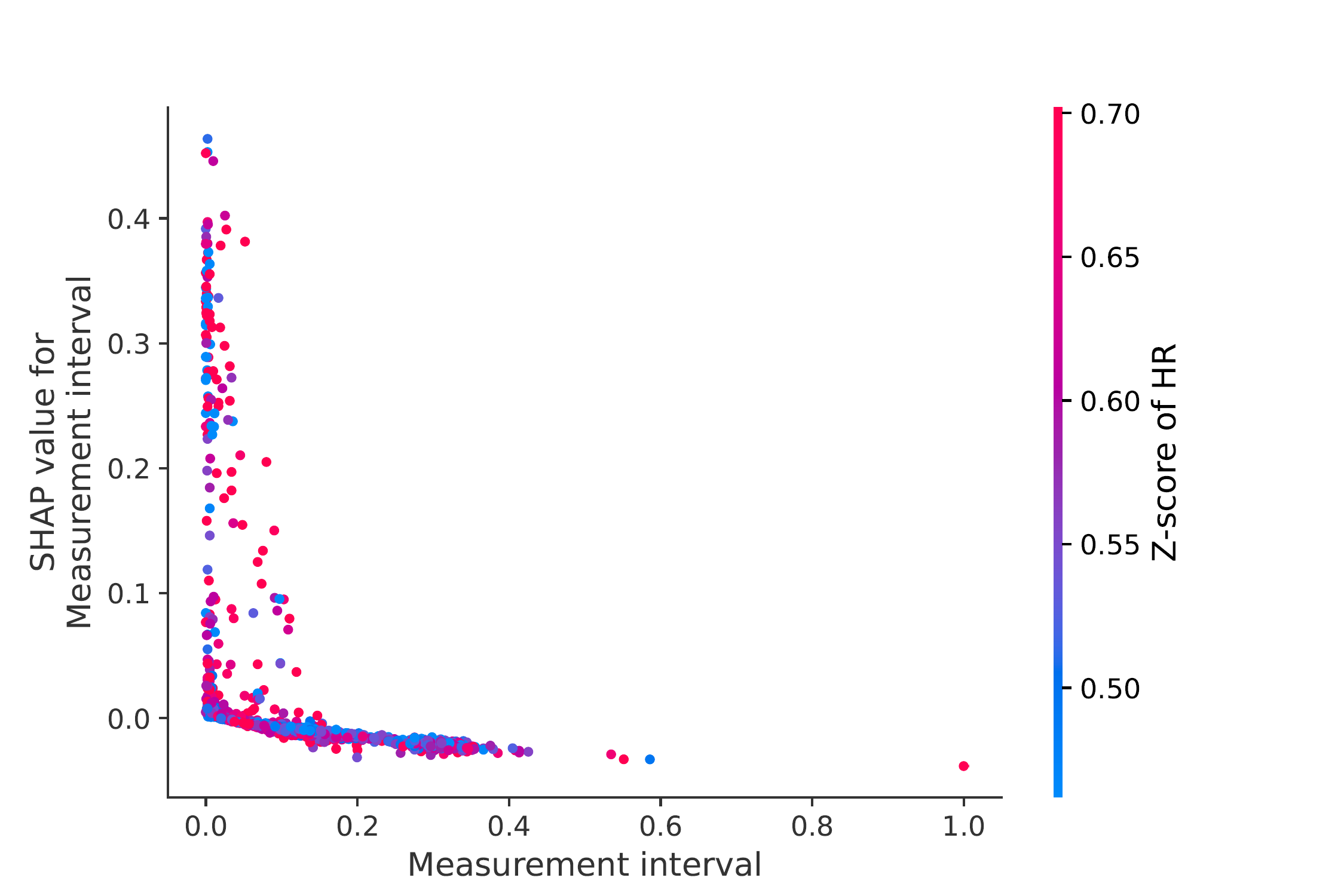


**Figure S4**. SHAP value (impact on the model output) relationship between measurement interval and z-score of HR.

SHAP = shapley additive explanations, HR = heart rate


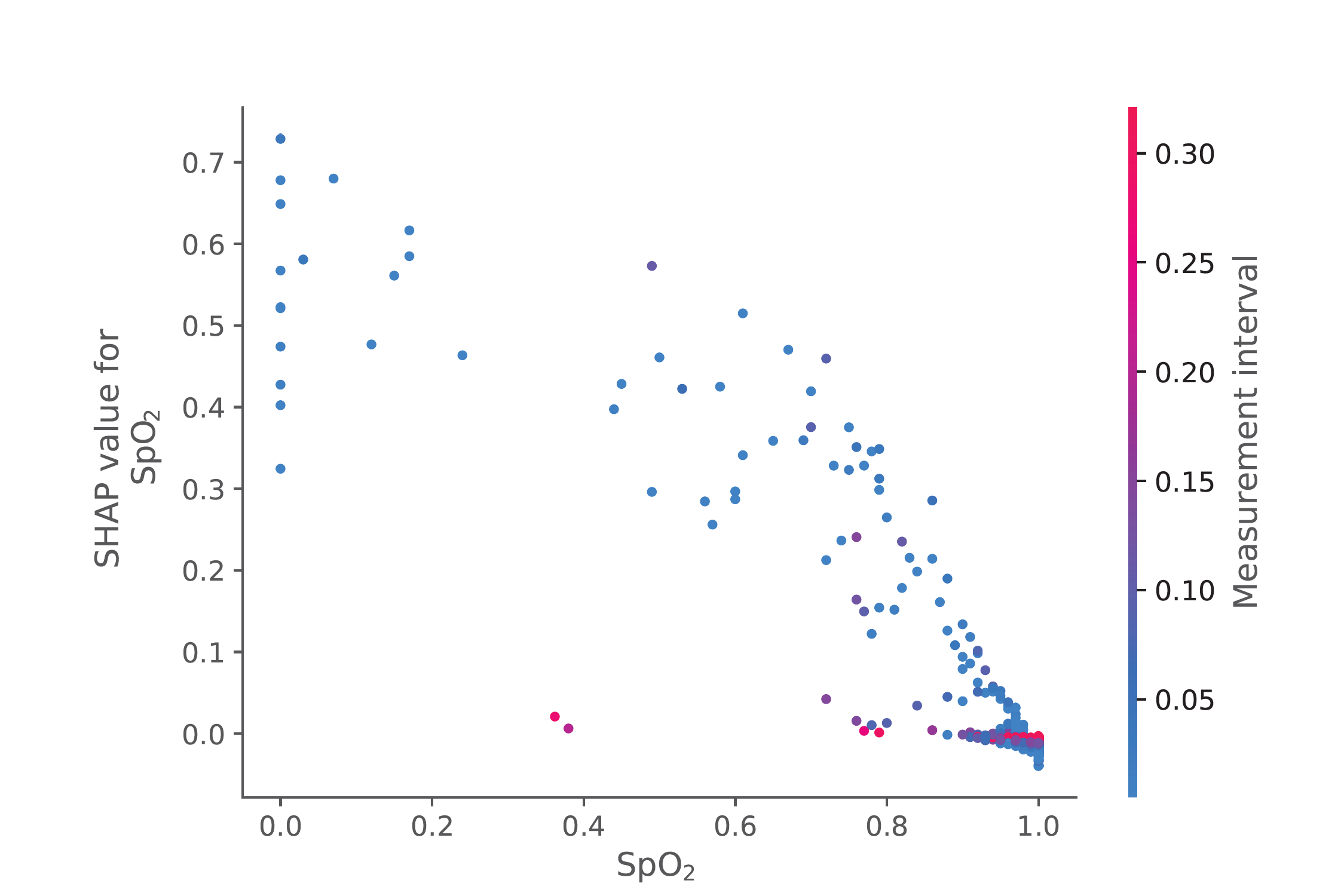


**Figure S5**. SHAP value (impact on the model output) relationship between SpO_2_ and measurement interval.

SHAP = shapley additive explanations, SpO_2_ = oxygen saturation


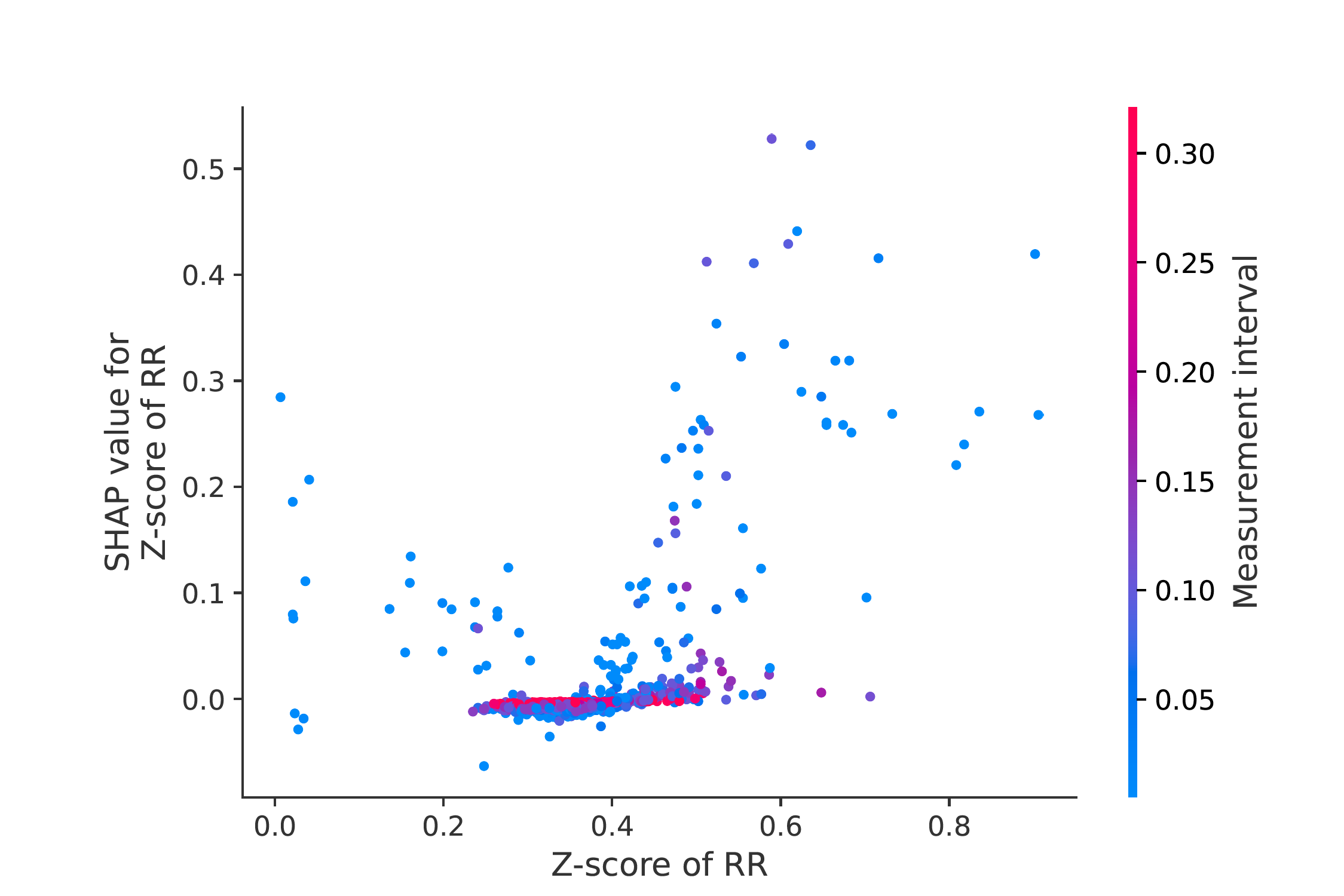


**Figure S6**. SHAP value (impact on the model output) relationship between z-score of RR and measurement interval.

SHAP = shapley additive explanations, RR = respiratory rate


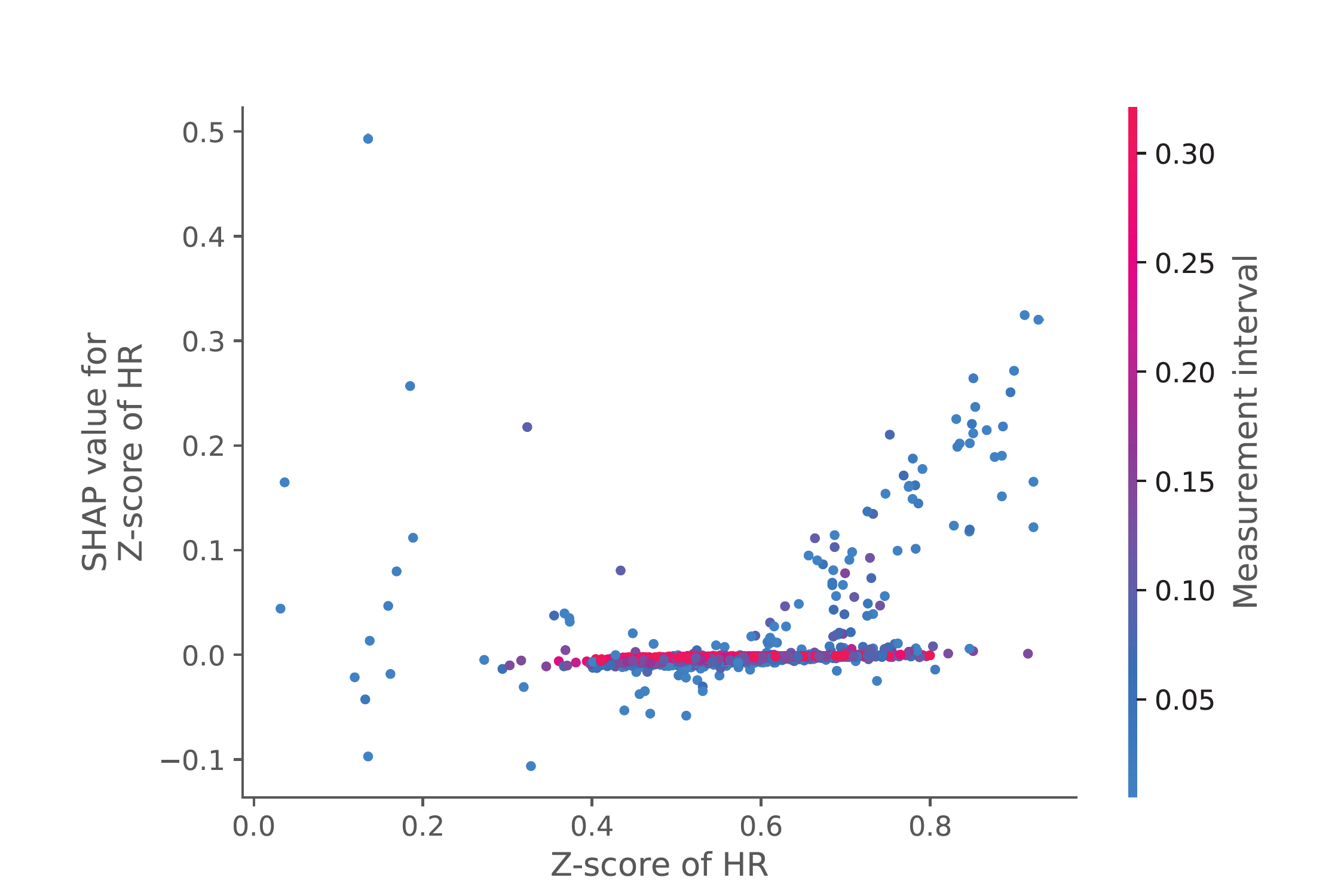


**Figure S7**. SHAP value (impact on the model output) relationship between z-score of HR and measurement interval.

SHAP = shapley additive explanations, HR = heart rate


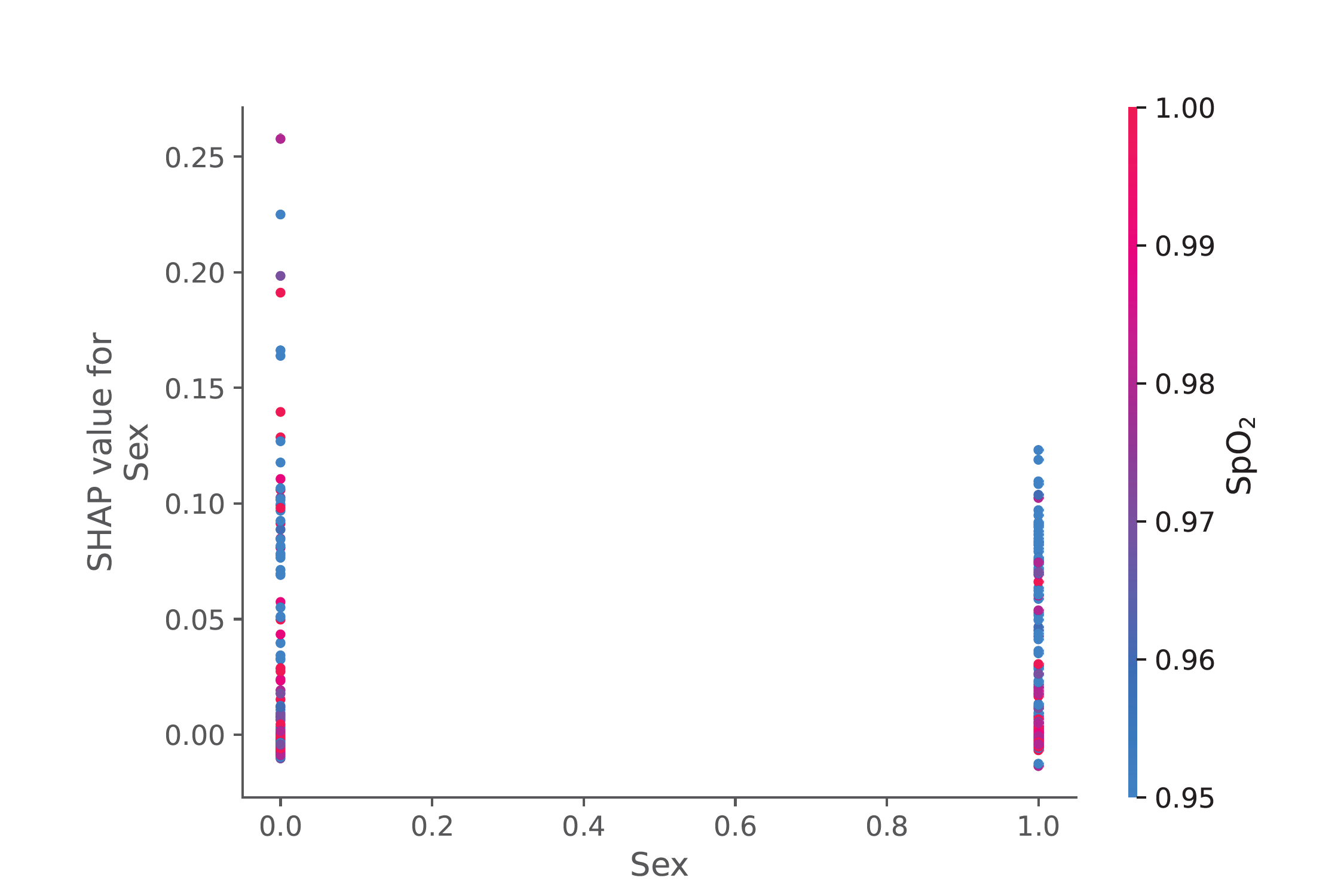


**Figure S8**. SHAP value (impact on the model output) relationship between sex and SpO_2_.

SHAP = shapley additive explanations, SpO_2_ = oxygen saturation


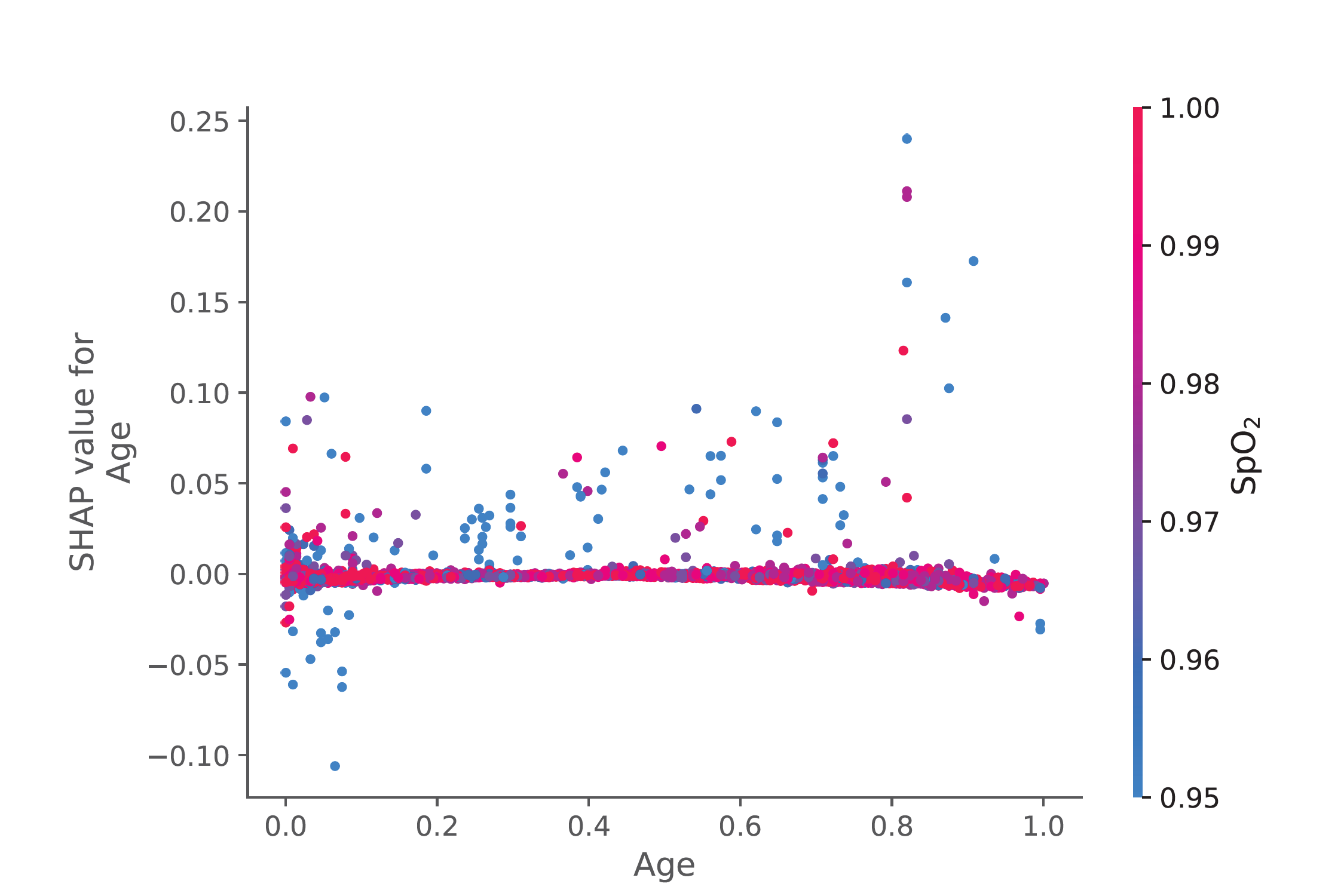


**Figure S9**. SHAP value (impact on the model output) relationship between age and SpO_2_.

SHAP = shapley additive explanations, SpO_2_ = oxygen saturation


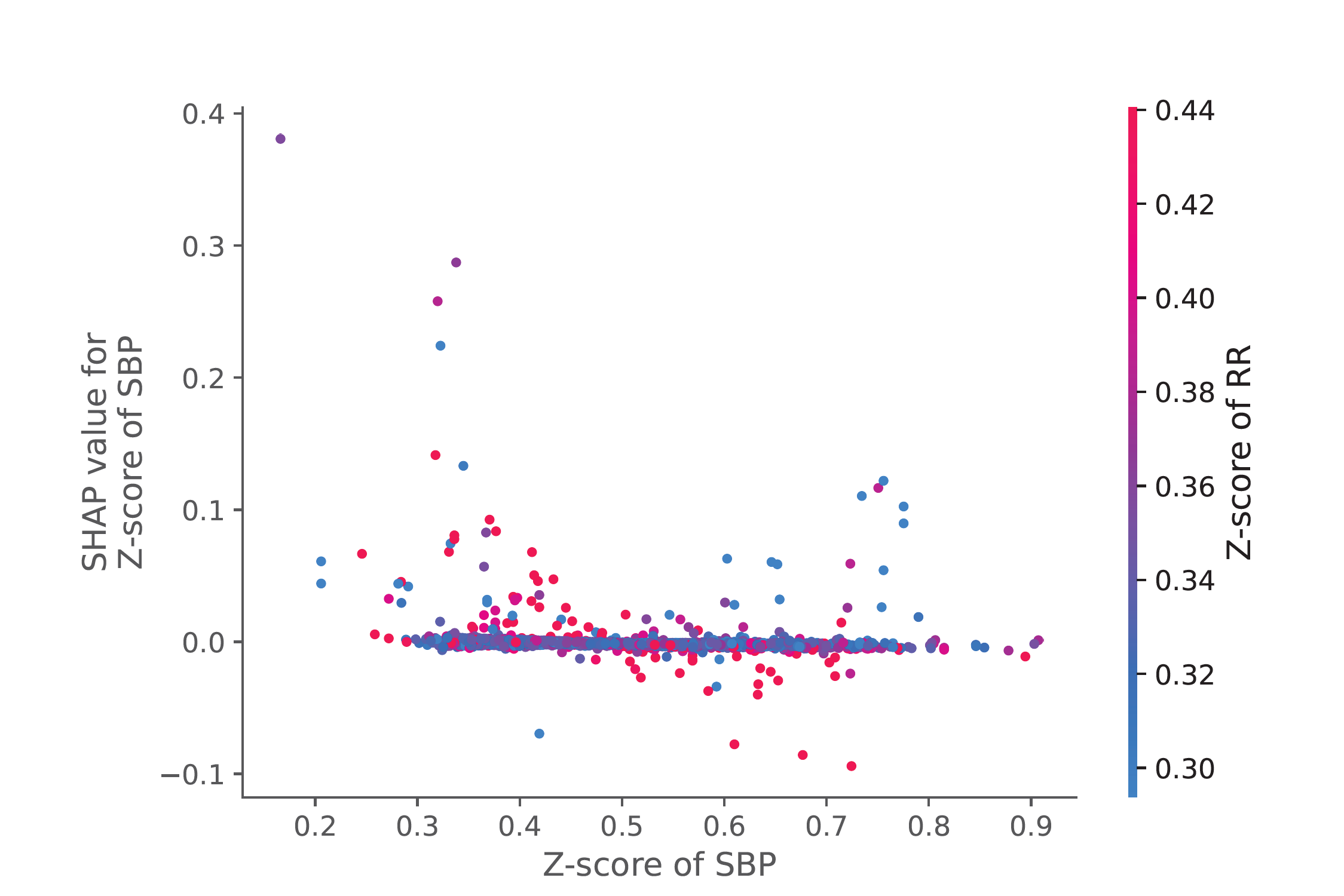


**Figure S10**. SHAP value (impact on the model output) relationship between z-score of SBP and z-score of RR.

SHAP = shapley additive explanations, SBP = systolic blood pressure, RR = respiratory rate


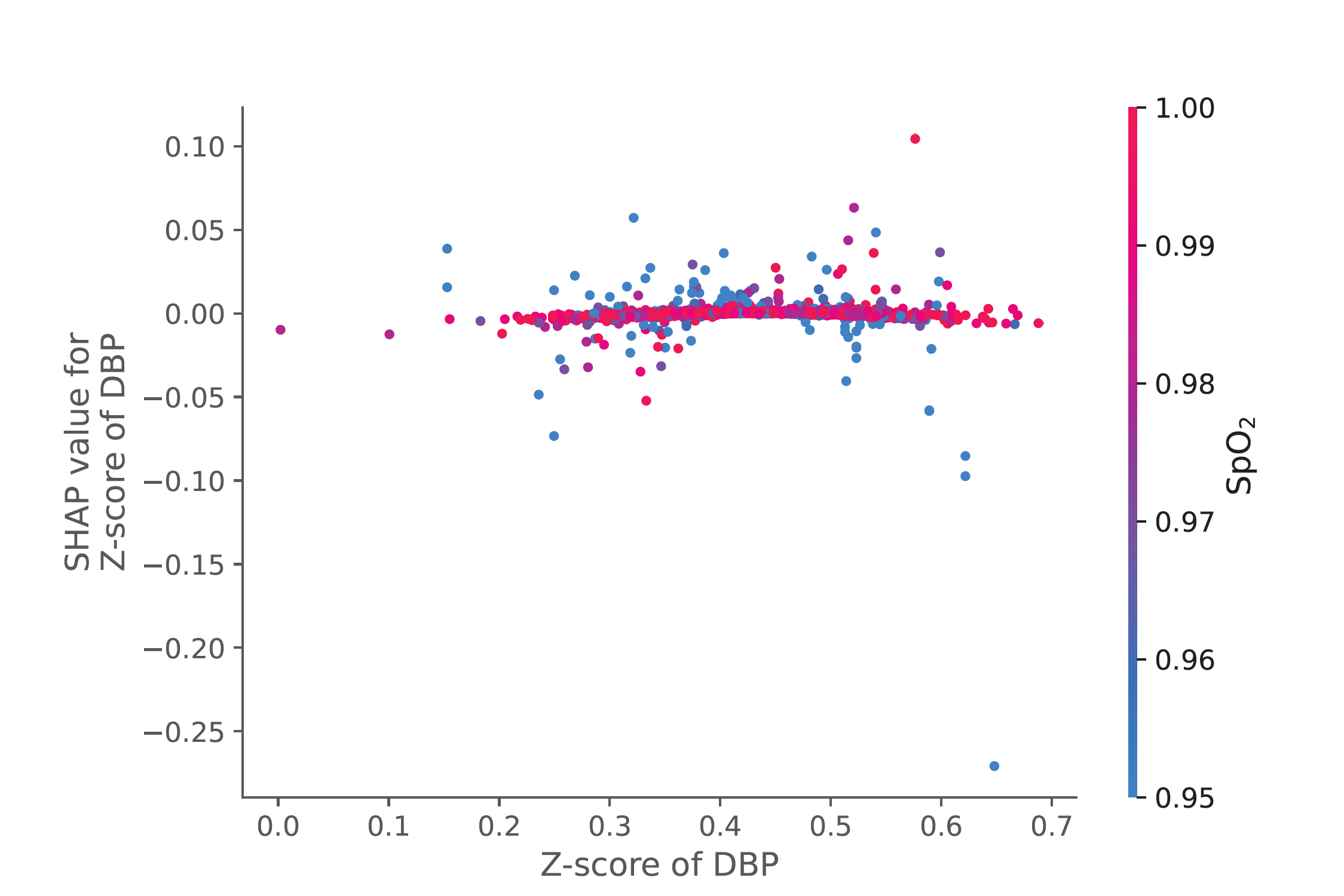


**Figure S11**. SHAP value (impact on the model output) relationship between z-score of DBP and SpO_2_.

SHAP = shapley additive explanations, DBP = diastolic blood pressure, SpO_2_ = oxygen saturation


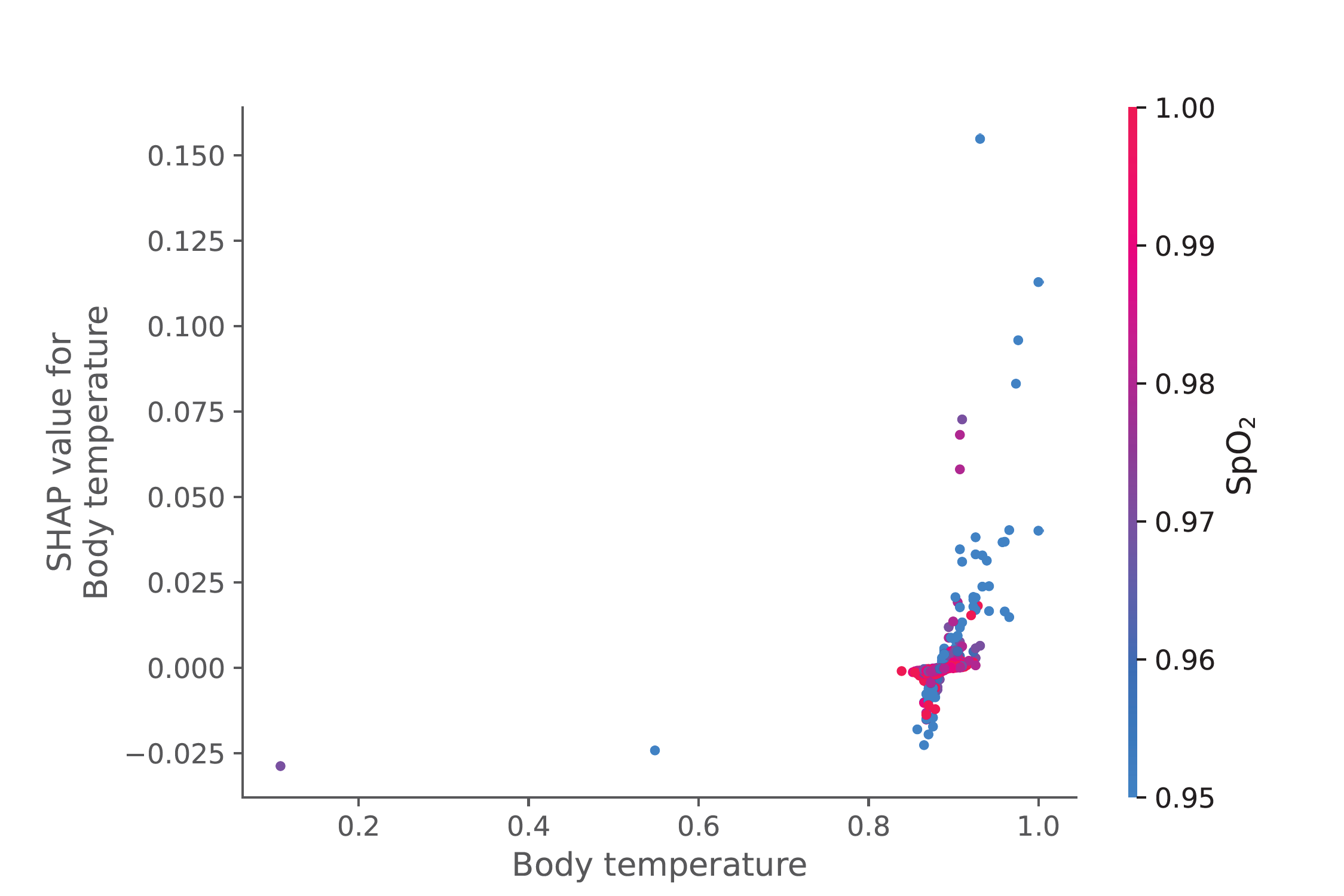


**Figure S12**. SHAP value (impact on the model output) relationship between body temperature and SpO_2_.

SHAP = shapley additive explanations, SpO_2_ = oxygen saturation
